# Psychiatric comorbidity and risk of premature mortality and suicide among those with chronic respiratory diseases, cardiovascular diseases, and diabetes in Sweden: A nationwide matched cohort study of over 1 million patients and their unaffected siblings

**DOI:** 10.1371/journal.pmed.1003864

**Published:** 2022-01-27

**Authors:** Amir Sariaslan, Michael Sharpe, Henrik Larsson, Achim Wolf, Paul Lichtenstein, Seena Fazel

**Affiliations:** 1 Department of Psychiatry, University of Oxford, Warneford Hospital, Oxford, United Kingdom; 2 Department of Medical Epidemiology and Biostatistics, Karolinska Institutet, Stockholm, Sweden; 3 School of Medical Sciences, Örebro University, Örebro, Sweden; University Hospital Hamburg, GERMANY

## Abstract

**Background:**

Persons with noncommunicable diseases have elevated rates of premature mortality. The contribution of psychiatric comorbidity to this is uncertain. We aimed to determine the risks of premature mortality and suicide in people with common noncommunicable diseases, with and without psychiatric disorder comorbidity.

**Methods and findings:**

We used nationwide registries to study all individuals born in Sweden between 1932 and 1995 with inpatient and outpatient diagnoses of chronic respiratory diseases (*n =* 249,825), cardiovascular diseases (*n* = 568,818), and diabetes (*n* = 255,579) for risks of premature mortality (≤age 65 years) and suicide until 31 December 2013. Patients diagnosed with either chronic respiratory diseases, cardiovascular diseases, or diabetes were compared with age and sex-matched population controls (*n =* 10,345,758) and unaffected biological full siblings (*n* = 1,119,543). Comorbidity with any psychiatric disorder, and by major psychiatric categories, was examined using diagnoses from patient registers. Associations were quantified using stratified Cox regression models that accounted for time at risk, measured sociodemographic factors, and unmeasured familial confounders via sibling comparisons. Within 5 years of diagnosis, at least 7% (range 7.4% to 10.8%; *P* < 0.001) of patients with respiratory diseases, cardiovascular diseases, or diabetes (median age at diagnosis: 48 to 54 years) had died from any cause, and 0.3% (0.3% to 0.3%; *P* < 0.001) had died from suicide, 25% to 32% of people with these medical conditions had co-occurring lifetime diagnoses of any psychiatric disorder, most of which antedated the medical diagnosis. Comorbid psychiatric disorders were associated with higher all-cause mortality (15.4% to 21.1%) when compared to those without such conditions (5.5% to 9.1%). Suicide mortality was also elevated (1.2% to 1.6% in comorbid patients versus 0.1% to 0.1% without comorbidity). When we compared relative risks with siblings without noncommunicable diseases and psychiatric disorders, the comorbidity with any psychiatric disorder was associated with substantially increased mortality rates (adjusted HR range: aHR_CR_ = 7.2 [95% CI: 6.8 to 7.7; *P* < 0.001] to aHR_CV_ = 8.9 [95% CI: 8.5 to 9.4; *P* < 0.001]). Notably, comorbid substance use disorders were associated with a higher mortality rate (aHR range: aHR_CR_ = 8.3 [95% CI: 7.6 to 9.1; *P* < 0.001] to aHR_CV_ = 9.9 [95% CI: 9.3 to 10.6; *P* < 0.001]) than depression (aHR range: aHR_CR_ = 5.3 [95% CI: 4.7 to 5.9; *P* < 0.001] to aHR_CV_ = 7.4 [95% CI: 7.0 to 7.9; *P* < 0.001]), but risks of suicide were similar for these 2 psychiatric comorbidities.

One limitation is that we relied on secondary care data to assess psychiatric comorbidities, which may have led to missing some patients with less severe comorbidities. Residual genetic confounding is another limitation, given that biological full siblings share an average of half of their cosegregating genes. However, the reported associations remained large even after adjustment for shared and unmeasured familial confounders.

**Conclusions:**

In this longitudinal study of over 1 million patients with chronic health diseases, we observed increased risks of all-cause and suicide mortality in individuals with psychiatric comorbidities. Improving assessment, treatment, and follow-up of people with comorbid psychiatric disorders may reduce the risk of mortality in people with chronic noncommunicable diseases.

## Abstract 2

In a nationwide cohort study, Amir Sariaslan and colleagues study the risk of premature mortality and suicide among those with noncommunicable diseases and psychiatric comorbidities compared to unaffected populations controls and siblings.

## Author summary

**Why was this study done?:**

- Noncommunicable diseases are a global public health challenge accounting for an excess of 40 million deaths annually.
- Comorbid psychiatric disorders have been identified as potential risk markers for premature mortality in patients with noncommunicable diseases.
- There is uncertainty about the extent and the nature of the association between psychiatric comorbidities and premature mortality.
- As most of the research has focused on comorbid depression as a risk marker and all-cause mortality as outcome, less is known about other psychiatric comorbidities and cause-specific mortality, including death by suicide.

**What did the researchers do and find?:**

- We used national registers in Sweden to investigate over 1 million patients born between 1932 and 1995 and diagnosed with chronic respiratory diseases, cardiovascular diseases, and diabetes.
- More than 7% of the patients died of any cause within 5 years, and 0.3% died from suicide. These risks were more than doubled in patients with psychiatric comorbidities compared to those without such comorbidities.
- Most of the psychiatric comorbidities were identified and diagnosed before the noncommunicable diseases.
- By comparing each of the patients with their unaffected siblings, we accounted for familial risk factors that were shared between the siblings (e.g., genetic and childhood environmental risk factors).
- We found that psychiatric comorbidity was consistently associated with elevated rates of premature mortality and suicide in the sibling comparison analyses and persisted following additional adjustments for sociodemographic factors and body mass index (BMI).

**What do these findings mean?:**

- Identification and treatment of co-occurring substance use disorders and depression in people admitted to general hospitals for chronic health conditions may improve mortality and morbidity in these patients.
- Public health initiatives can consider how to improve detection and management of comorbid psychiatric conditions in primary care.
- New models for the delivery of more integrated services for physical health, psychiatry, and substance use disorders need investigation as part of wider measures to reduce mortality in noncommunicable diseases.

## Introduction

Noncommunicable diseases are one of the most important public health challenges worldwide. An estimated 41 million deaths were caused by noncommunicable diseases worldwide in 2016, most of which (23 million deaths) were specifically attributed to 3 prevalent conditions: cardiovascular diseases, chronic respiratory diseases, and diabetes [1]. This mortality burden is likely to increase in the coming decades, particularly driven by increasing rates in low- and middle-income countries [2,3]. Reducing premature mortality is thus a key challenge for national healthcare strategies and is included in the UN’s Sustainable Development Goals.

Comorbid psychiatric disorders are a potentially modifiable risk factor for premature death in people with one or multiple noncommunicable diseases [4,5]. Depression was reported in 9% of people with diabetes, 15% of people with angina in a survey of nearly 250,000 participants across 60 countries [6], and 15% of people with chronic obstructive pulmonary disease (COPD) in a large US survey [7]. Comorbid depression is associated with poor prognosis and elevated mortality in acute coronary syndrome [8], diabetes [9], COPD [10], and other chronic diseases [11]. However, the robustness and precision of the mortality risk is uncertain. Previous work has not adjusted for important confounds, such as genetic and sociodemographic factors [12]. In addition, the contribution of other common comorbid psychiatric and substance use disorders to mortality risks is uncertain [4,13]. In particular, the association of psychiatric comorbidity with specific-cause mortality, particularly suicide, is not known as previous studies have been insufficiently powered.

A key limitation of previous work is that it has not accounted for familial factors that may increase risks of adverse health outcomes. Noncommunicable diseases (e.g., COPD [14,15], heart disease [16,17], and diabetes [18]), psychiatric disorders [19], and premature mortality (including suicide risk) [20] all aggregate in families. The same genetic and early environmental risk factors that increase risk of mortality could potentially also increase likelihood of chronic health conditions and comorbid psychiatric disorders. Consequently, causal inferences are not possible without careful adjustment for such factors suggesting that previous research may have overestimated associations.

Therefore, in this study, we have linked a number of high-quality Swedish national registers to report rates of premature mortality (≤age 65) and suicide for 3 common noncommunicable diseases (e.g., chronic respiratory diseases, cardiovascular diseases, and diabetes) according to psychiatric comorbidity. By investigating over 1 million patients with these noncommunicable diseases over a 41-year period, we aimed to provide precise estimates of mortality risks. In contrast to much previous work that has been limited to a single baseline measurement of psychiatric comorbidities, the prospective nature of the current study allowed us to obtain a comprehensive measure of preexisting psychiatric disorders spanning up to multiple decades. In addition, we examined biological full siblings unaffected by these chronic conditions to assess the possible role of unmeasured genetic and early environmental confounders.

## Methods

This study was approved by the Regional Ethics Committee at the Karolinska Institutet (2013/5:8). Data were merged and pseudonymized by an independent government agency, and, after merging, the code linking the personal identification numbers to the new case numbers was destroyed immediately. Therefore, informed consent from individual patients was not required. The study did not have a prospective analysis plan.

### Study setting

We linked longitudinal, nationwide population-based registries in Sweden: the National Patient Register (held at the National Board of Health and Welfare), the National Censuses from 1970 and 1990 (Statistics Sweden), the Multi-Generation, and the National Cause-of-Death Registers (Statistics Sweden). The Multi-Generation Register connects each person born in Sweden in 1932 or later and ever registered as living in Sweden after 1960 to their parents [21]. In Sweden, every resident has a unique personal identifier used in all national registers, thereby enabling accurate data linkage [22]. We selected the cohort of all individuals born 1932 to 1995, which was followed up during 1973 to 2013. This study is reported as per the Strengthening the Reporting of Observational Studies in Epidemiology (STROBE) guideline (S1 Checklist).

### Individuals with noncommunicable diseases

We chose 3 noncommunicable diseases—chronic respiratory diseases, cardiovascular diseases, and diabetes (type 1 and 2)—because of their high prevalence, public health importance, and diagnostic validity in Swedish healthcare registers. We identified individuals with diagnosed chronic respiratory diseases, cardiovascular diseases, and diabetes over the age of 18 from the Patient Register, which includes individuals admitted to any hospital (national coverage from 1973) or having outpatient appointments with specialist physicians (since 2001) [21]. Cases had at least 1 patient episode (primary, secondary, or additional diagnoses) for these 3 conditions according to the International Classification of Diseases (ICD) (S1 Table for diagnostic codes). Given that specific diagnoses for diabetes subtypes (e.g., type 1 and 2) were first introduced in the 10th revision of ICD, we were only able to identify such patients after 1 January 1997. For validity of the cardiovascular diagnoses, there have been 11 such studies [23], with positive predictive values (PPVs) ranging from 69% (nonfatal stroke) to 100% (myocardial infarction) for a register diagnosis predicting a clinically based review of all medical records. In diabetes, the PPV is reported as 79% [24]. For chronic respiratory diseases, there are 2 validity studies for asthma with PPVs of 89% [25] and 93% [24].

### Outcome measures

We defined premature mortality as death before the age of 66 years in line with previous work [26]. Thus, mortality data were retrieved for individuals who died before age 66, between 1973 and 2013. The Cause of Death register is based on death certificates and covers over 99% of all deaths [27]. In line with previous work [28], uncertain suicides were included as suicides since their exclusion could lead to underestimation of the rate [29].

### Unaffected general population and sibling controls

For each case and condition, up to 10 general population controls without that particular medical condition were matched individually by birth year and sex, which means that there are inherently adjusted for age and sex. The controls had to be alive at the time of the matching date. In order to assess familial confounding, we additionally used sibling controls. Using the Multi-Generation Register, we identified patients who also had one or more full siblings without the studied noncommunicable disease, who were alive and residents of Sweden at the time of the matching date. Patients were compared for risk of premature death and suicide with unaffected full siblings of both sexes. Thus, all potential sibling pairs were investigated with adjustments for sex and birth year. Flow charts of the samples are presented in S1 Fig.

### Psychiatric covariates

Psychiatric disorders were identified using a similar approach as the noncommunicable diseases by using the Patient Register over 1973 to 2013 and using ICD diagnoses. Data were extracted for all cases and controls on all inpatient and outpatient diagnoses with principal or comorbid diagnoses of depression and related mood disorders, alcohol or drug abuse or dependence, and any psychiatric disorder (i.e., any ICD-10 diagnostic code F00 to F99, which includes these 2 former diagnostic groups but also severe mental illnesses [e.g., schizophrenia and bipolar disorder], anxiety disorders, and personality disorders) (S1 Table for ICD codes). These register-recorded diagnoses have been shown to be valid with fair to moderate agreement with clinician-based diagnoses reported for depression (κ of 0.32; 88% full agreement) [30] and for comorbid substance use disorder in schizophrenia (κ = 0.37, 68% full agreement) [31].

### Additional sociodemographic confounders

Annual disposable income data were collected from the Income and Taxation Register (1968 to 1989) and the Integrated Database for Labour Market Research (1990 to 2013). We standardized income measures by year and calculated the median income (before first diagnosis), divided into deciles, and dichotomized into lowest decile versus top 9 deciles. Single marital status was defined at first diagnosis. If data were missing, we chose the first available measurement prior to diagnosis. Migrant background was defined as either being born outside of the Nordic countries (e.g., Denmark, Finland, Iceland, Norway, and Sweden) or having at least 1 parent fulfilling the same criteria. Approximately 2.7% of the full sample lacked income data and were excluded.

### Analytic approach

We initially estimated sex-specific risks of premature death and suicide following a diagnosis of either chronic respiratory diseases, cardiovascular diseases, or diabetes, using matched unrelated or sibling controls [31,32]. We fitted stratified Cox regression models to matched groups, thus allowing for varying baseline hazards across the groups. This implies that the comparisons, expressed as hazard ratios, were made within each group, thereby controlling for the matched characteristics. We followed up patients and controls from the same point in time, i.e., from first diagnosis of the noncommunicable disease, until they either migrated, died, turned 65 years of age or at the end point of the study (31 December 2013).

The stratified Cox regression model assumes proportional hazards, namely that the hazard ratios remain constant across time. Although the mortality rates were consistently larger in the patients compared to the controls throughout the follow-up period, we found by examining Schoenfeld residuals that the proportional hazards assumption was not supported in many models [33], meaning that the magnitude of the hazard ratios varied across time. It is therefore important to interpret the presented hazard ratios as weighted averages of the time-varying hazard ratios across the follow-up period [34].

When using siblings as controls, we were able to account for all time-constant unmeasured familial confounders shared between the siblings, including about half of their cosegregating genes and shared childhood environments. We fitted 2 sets of sibling comparison models: crude models were, similar to the models using population controls, only adjusted for sex and birth year, while the adjusted models additionally accounted for low income and single marital status. We chose the 2 latter potential confounders on theoretical grounds, based on related work in epilepsy [35] and head injury [36], and tested if they were each associated with case status and outcome measures, respectively [37]. The extent to which the sibling control estimates were attenuated relative to the population control estimates is an indicator of the influence of the unmeasured familial confounders.

We further investigated whether being diagnosed with comorbid noncommunicable diseases was associated with elevated mortality risks by refitting the models to a subset of patients who had been diagnosed with 2 or all 3 of the noncommunicable diseases and compared them with a subset of their population and sibling controls who had not been diagnosed with any of the 3 diseases.

To quantify the contributions of the comorbid psychiatric disorders, we reran separate sibling comparison models for each combination of noncommunicable disease and comorbid condition (e.g., any psychiatric disorder, depression, and substance use disorder). The models also included an interaction term that estimated their multiplicative effects. We only considered preexisting psychiatric disorders because post-onset psychiatric disorders could potentially be caused by a combination of the stressors associated with being diagnosed with a noncommunicable disease and surveillance bias (e.g., patients being referred to psychiatric services by their physicians). Together, these factors could cause reverse causation bias. To aid interpretation, we applied linear combinations of the parameters that specifically quantified the joint contributions of the noncommunicable diseases and the comorbid psychiatric disorders versus the contributions of the noncommunicable diseases without the comorbid conditions. We omitted interaction terms that were not statistically significant (*P* > 0.05) in these estimations.

In complementary sensitivity analyses, we explored whether lifetime psychiatric disorders were differentially associated with premature mortality compared with preexisting measures of the same disorders. The advantage of this set of analyses was that potentially undiagnosed disorders could be included, but this came at the cost of both elevated ascertainment bias for psychiatric disorders and immortal time bias (i.e., conditioning the exposure on an event that occurred during the follow-up, such as being diagnosed with a psychiatric disorder, makes the cases “immortal” during the time until the event has occurred unlike the controls who may die during the same period) [38]. We also examined whether further stratification into specific diagnoses of diabetes subtypes (e.g., type 1 versus 2) in a subset of the patients diagnosed after 1996 (n_cases_ = 63,216; n_controls_ = 116,702) and substance use disorders in the full samples impacted the findings.

During the review process, we were asked to conduct 3 additional sensitivity analyses. First, we stratified the patients across those who had their first presentation of the noncommunicable diseases in inpatient care settings versus specialist outpatient settings. Second, to test for the confounding role of obesity, we were able to examine body mass index (BMI) (as a proxy) in male conscripts who underwent physical health examinations at age of 18. Third, we excluded individuals diagnosed with multiple noncommunicable diseases. Overall, analyses were conducted between 17 February 2020 and 6 September 2021. We used Stata 17 MP for the Cox regression models (*stcox* and *lincom* commands) and R 4.1.1 for the time-specific cumulative mortality risks (*cmprsk*::*cuminc()*).

## Results

Our sample included a total of 1,074,222 patients, of which 249,825 had been diagnosed with chronic respiratory diseases, 568,818 with cardiovascular diseases, and 255,579 with diabetes (Table 1). The patients and population controls were followed up for 92.9 million person-years and for an average of 8.1 years (SD = 7.2) per patient. Around 1 in 10 men diagnosed with any of the 3 noncommunicable diseases died within 5 years, ranging from 9.5% (95% CI: 9.3% to 9.7%; *P* < 0.001) to 11.0% (95% CI: 10.9% to 11.1%; *P* < 0.001), and 0.4% died from suicide (Table 2). In women with noncommunicable diseases, we observed larger heterogeneity in the 5-year all-cause mortality risks, ranging from 5.9% (95% CI: 5.8% to 6.0%; *P*<0.001) to 10.5% (95% CI: 10.4% to 10.7%; *P* < 0.001), and up to 0.3% died of suicide (Table 2). Compared to population controls, men with noncommunicable diseases were 4 times more likely to have died from any cause (crude hazard ratio [cHR] range: cHR_Cardiovascular diseases [CV]_ = 3.7 95% CI: 3.7 to 3.7; *P* < 0.001 to cHR_Diabetes [D]_ = 4.4; 4.3 to 4.4; *P* < 0.001) and had 2-fold increased risk of suicide death (cHR range: cHR_CV_ = 1.9 [95% CI: 1.8 to 2.0; *P* < 0.001] to cHR_Chronic respiratory diseases [CR]_ = 2.4 [95% CI: 2.2 to 2.6; *P* < 0.001]; Table 3). Women with noncommunicable diseases had a 4- to 5-fold increased risk of premature death compared to population controls (cHR range: cHR_CR_ = 4.2 [95% CI: 4.1 to 4.3; *P* < 0.001] to cHR_CV_ = 5.4 [95% CI: 5.4 to 5.5; *P* < 0.001]), and their suicide risks were elevated (cHR_D_ = 2.3 [95% CI: 2.0 to 2.6; *P* < 0.001] to cHR_CR_ = 3.3 [95% CI: 3.0 to 3.7; *P* < 0.001]; Table 3). We observed similar results on the population level when we examined premature mortality (cHR range: 4.0 to 4.6; *P* < 0.001) and suicide (cHR range: 2.1 to 2.7; *P* < 0.001) as outcome (Table 3). The crude sibling comparison models indicated that these associations were slightly confounded by unmeasured familial factors (cHR_Premature mortality [PM]_ range: 3.3 to 4.1; *P* < 0.001; cHR_Suicide [S]_ range: 2.0 to 2.4; *P* < 0.001). Additional adjustments for sociodemographic factors contributed negligibly to explaining these differences (adjusted hazard ratio [aHR], aHR_PM_ range = 3.3 to 4.0; *P* < 0.001; aHR_S_ range: 2.0 to 2.4; *P* < 0.001).

**Table 1:** Baseline sociodemographic information for cohorts of patients and comparison groups.

	Patients	General population controls	Patients with unaffected siblings	Unaffected sibling controls
***Chronic respiratory diseases***				
Total *n*	249,825	2,378,292	152,320	263,250
Premature mortality (suicide), *n*	25,078 (1,136)	67,108 (4,204)	13,362 (635)	9,651 (642)
Person-years at risk (mean)	1,911,925 (7.7)	18,852,037 (7.9)	1,212,637 (8.0)	2,409,063 (9.2)
Male sex, *n* (percent)	105,298 (42.1%)	1,004,031 (42.2%)	63,938 (42.0%)	133,757 (50.8%)
Single status, *n* (percent)	88,682 (35.5%)	792,842 (33.3%)	60,549 (39.8%)	96,449 (36.6%)
Migrant background, *n* (percent)	28,868 (11.6%)	261,449 (11.0%)	3,226 (2.1%)	6,547 (2.5%)
Low income, *n* (percent)	24,933 (10.0%)	232,993 (9.8%)	17,402 (11.4%)	17,648 (6.7%)
Median age at diagnosis, years (IQR)	48 (34–58)	-	46 (33–57)	-
Median age at death, years (IQR)	59 (53–63)	60 (54–63)	59 (54–63)	59 (53–63)
***Cardiovascular diseases***				
Total *n*	568,818	5,500,341	332,813	602,005
Premature mortality (suicide), *n*	81,530 (2,457)	221,466 (12,419)	41,040 (1,339)	25,611 (1,423)
Person-years at risk (mean)	4,192,389 (7.4)	44,522,629 (8.1)	2,584,808 (7.8)	5,846,550 (9.7)
Male sex, *n* (percent)	363,282 (63.9%)	3,514,535 (63.9%)	214,296 (64.4%)	297,749 (49.5%)
Single status, *n* (percent)	138,885 (24.4%)	1,318,339 (24.0%)	93,437 (28.1%)	168,218 (27.9%)
Migrant background, *n* (percent)	54,858 (9.6%)	496,496 (9.0%)	4,301 (1.3%)	8,087 (1.3%)
Low income, *n* (percent)	63,936 (11.2%)	542,081 (9.9%)	35,521 (10.7%)	56,992 (9.5%)
Median age at diagnosis, years (IQR)	54 (45–60)	-	53 (43–59)	-
Median age at death, years (IQR)	58 (51–62)	61 (56–63)	58 (52–62)	59 (55–63)
***Diabetes***				
Total *n*	255,579	2,467,125	140,710	254,288
Premature mortality (suicide), *n*	39,119 (1177)	96,996 (5923)	19,406 (609)	11,359 (680)
Person-years at risk (mean)	2,034,631 (8.0)	21,419,226 (8.7)	1,220,082 (8.7)	2,653,295 (10.4)
Male sex, *n* (percent)	157,365 (61.6%)	1,522,745 (61.7%)	87,847 (62.4%)	127,906 (50.3%)
Single status, *n* (percent)	70,500 (27.6%)	595,796 (24.1%)	45,719 (32.5%)	73,877 (29.1%)
Migrant background, *n* (percent)	36,820 (14.4%)	218,388 (8.9%)	1,989 (1.4%)	4,006 (1.6%)
Low income, *n* (percent)	37,195 (14.6%)	235,036 (9.5%)	15,232 (10.8%)	23,222 (9.1%)
Median age at diagnosis, years (IQR)	54 (44–60)	-	53 (42–60)	-
Median age at death, years (IQR)	58 (51–62)	60 (55–63)	58 (52–62)	59 (54–63)

IQR, interquartile range.

Low income was defined as the bottom decile of the median disposable income accumulated until the end of the year before the onset of the noncommunicable disease and during the equivalent period for the controls.

**Table 2:** Five-year cumulative risks (percentage [95% confidence intervals]) of premature mortality and suicide across noncommunicable diseases and stratified by sex.

	Premature mortality		Suicide	
	Patients	Controls	Patients	Controls
**All individuals**				
Chronic respiratory diseases	7.4% [7.3%; 7.5%]	1.6% [1.6%; 1.6%]	0.3% [0.3%; 0.4%]	0.1% [0.1%; 0.1%]
Cardiovascular diseases	10.8% [10.7%; 10.9%]	2.2% [2.2%; 2.2%]	0.3% [0.3%; 0.4%]	0.1% [0.1%; 0.1%]
Diabetes	9.4% [9.2%; 9.5%]	2.1% [2.1%; 2.2%]	0.3% [0.3%; 0.3%]	0.1% [0.1%; 0.1%]
**Men**				
Chronic respiratory diseases	9.5% [9.3%; 9.7%]	2.2% [2.1%; 2.2%]	0.4% [0.4%; 0.5%]	0.2% [0.2%; 0.2%]
Cardiovascular diseases	11.0% [10.9%; 11.1%]	2.6% [2.5%; 2.6%]	0.4% [0.3%; 0.4%]	0.2% [0.2%; 0.2%]
Diabetes	10.3% [10.1%; 10.4%]	2.5% [2.5%; 2.5%]	0.4% [0.4%; 0.4%]	0.2% [0.2%; 0.2%]
**Women**				
Chronic respiratory diseases	5.9% [5.8%; 6.0%]	1.2% [1.2%; 1.2%]	0.3% [0.2%; 0.3%]	0.1% [0.1%; 0.1%]
Cardiovascular diseases	10.5% [10.4%; 10.7%]	1.6% [1.6%; 1.7%]	0.3% [0.2%; 0.3%]	0.1% [0.1%; 0.1%]
Diabetes	7.9% [7.7%; 8.1%]	1.6% [1.5%; 1.6%]	0.2% [0.2%; 0.2%]	0.1% [0.1%; 0.1%]

Notes: All of the estimates for the comparisons the between patients and controls were statistically significant (*P* < 0.001).

**Table 3:** Hazard ratios (HR) of premature mortality and suicide across noncommunicable diseases and stratified by sex.

		Patients versus population controls	Patients versus sibling controls	
		Crude	Crude	Adjusted
		HR [95% CI]	HR [95% CI]	HR [95% CI]
**Premature mortality**				
	**All individuals**			
	Chronic respiratory diseases	4.0 [4.0; 4.1]	3.3 [3.3; 3.4]	3.3 [3.2; 3.4]
	Cardiovascular diseases	4.1 [4.1; 4.2]	3.8 [3.8; 3.9]	3.8 [3.8; 3.9]
	Diabetes	4.6 [4.5; 4.6]	4.1 [4.0; 4.2]	4.0 [3.9; 4.1]
	**Men**			
	Chronic respiratory diseases	3.9 [3.8; 4.0]	3.2 [3.0; 3.4]	3.1 [2.9; 3.3]
	Cardiovascular diseases	3.7 [3.7; 3.7]	3.3 [3.2; 3.4]	3.3 [3.2; 3.4]
	Diabetes	4.4 [4.3; 4.4]	3.8 [3.6; 4.0]	3.7 [3.5; 3.9]
	**Women**			
	Chronic respiratory diseases	4.2 [4.1; 4.3]	3.3 [3.1; 3.6]	3.3 [3.1; 3.6]
	Cardiovascular diseases	5.4 [5.4; 5.5]	4.9 [4.6; 5.1]	4.8 [4.6; 5.1]
	Diabetes	5.0 [4.9; 5.1]	4.4 [4.1; 4.7]	4.4 [4.1; 4.7]
**Suicide**				
	**All individuals**			
	Chronic respiratory diseases	2.7 [2.6; 2.9]	2.4 [2.1; 2.8]	2.4 [2.1; 2.8]
	Cardiovascular diseases	2.1 [2.0; 2.2]	2.3 [2.1; 2.5]	2.2 [2.0; 2.5]
	Diabetes	2.1 [2.0; 2.2]	2.0 [1.8; 2.3]	2.0 [1.8; 2.3]
	**Men**			
	Chronic respiratory diseases	2.4 [2.2; 2.6]	2.2 [1.7; 2.7]	2.2 [1.8; 2.4]
	Cardiovascular diseases	1.9 [1.8; 2.0]	1.8 [1.6; 2.0]	2.1 [1.8; 2.3]
	Diabetes	2.1 [1.9; 2.2]	2.0 [1.6; 2.4]	2.3 [1.9; 2.7]
	**Women**			
	Chronic respiratory diseases	3.3 [3.0; 3.7]	2.5 [2.0; 3.3]	2.7 [2.1; 3.4]
	Cardiovascular diseases	3.2 [2.9; 3.5]	3.3 [2.6; 4.2]	3.3 [2.7; 4.1]
	Diabetes	2.3 [2.0; 2.6]	2.8 [2.0; 4.0]	3.1 [2.3; 4.2]

The estimates are based on stratified Cox regression models where groups of cases and controls (matched on sex and birth year) were treated as different strata. All biological full siblings were included as controls in the sibling models, which were adjusted for birth year and sex. The adjusted sibling comparison model additionally accounted for low income and single marital status. Migrant background was controlled for by the design as it does not vary within families. All of the estimates for the comparisons between the patients and either the population or sibling controls were statistically significant (*P* < 0.001).

### Comorbid psychiatric disorders

We found increased lifetime rates of psychiatric disorder in the sample, and specifically of substance use disorders, and depression (Table 4). Overall, 79,893 (32.0%) of patients with chronic respiratory diseases, 142,338 (25.0%) with cardiovascular diseases, and 72,126 (28.2%) of people with diabetes had a lifetime diagnosis of a comorbid psychiatric disorder, compared to approximately 16% of general population controls. Of the patients with chronic respiratory diseases who had a lifetime comorbid psychiatric disorder, we found that the psychiatric diagnoses preceded the chronic respiratory disease diagnoses in 65% (51,980/79,893; Table 4) of the cases. Equivalent estimates for patients with cardiovascular diseases and diabetes were slightly lower, ranging between 60% and 62%.

**Table 4:** Counts and prevalence rates of preexisting and lifetime psychiatric morbidity in cases and controls.

	Chronic respiratory diseases		Cardiovascular diseases		Diabetes	
	Patients, *n* (%) (*n* = 249,825)	Population controls, *n* (%) (*n* = 2,378,292)	Patients, *n* (%) (*n* = 568,818)	Population controls, *n* (%) (*n* = 5,500,341)	Patients, *n* (%) (*n* = 255,579)	Population controls, *n* (%) (*n =* 2,467,125)
*Any psychiatric disorder*						
Preexisting	51,980 (20.8%)	215,657 (9.1%)	85,142 (15.0%)	472,786 (8.6%)	44,466 (17.4%)	215,161 (8.7%)
Lifetime	79,893 (32.0%)	379,280 (15.9%)	142,338 (25.0%)	880 048 (16.0%)	72,126 (28.2%)	394,744 (16.0%)
*Depression*						
Preexisting	17,993 (7.2%)	72,777 (3.1%)	25,908 (4.6%)	149,195 (2.7%)	14,477 (5.7%)	69,437 (2.8%)
Lifetime	31,419 (12.6%)	140,224 (5.9%)	48,939 (8.6%)	295,455 (5.4%)	26,618 (10.4%)	135,661 (5.5%)
*Substance use disorders*						
Preexisting	20,888 (8.4%)	62,907 (2.6%)	34,387 (6.0%)	174,425 (3.2%)	17,821 (7.0%)	77,739 (3.2%)
Lifetime	28,734 (11.5%)	101,432 (4.3%)	49,471 (8.7%)	283,478 (5.2%)	25,993 (10.2%)	126,563 (5.1%)

Data are number (%) with psychiatric comorbidity. Any psychiatric disorder includes substance use diagnoses.

More patients with noncommunicable diseases and comorbid psychiatric disorders died during the first 5 years of follow-up (cumulative mortality risk range, all-cause mortality: 15.4% to 21.1%; suicide: 1.2% to 1.6%) than patients without psychiatric comorbidities (all-cause mortality: 5.5% to 9.1%; suicide: 0.1% to 0.1%) (Table 5). When examining specific psychiatric disorders, we observed that comorbid substance use disorders were associated with higher mortality risks (23.3% to 28.7%) than co-occurring depression (13.4% to 18.9%) but with no clear differences in suicide risks (1.5% to 2.4%; Tables 5 and S2 for 1-, 2-, and 5-year mortality estimates).

**Table 5:** Five-year cumulative premature mortality and suicide risks (percentage [95% confidence intervals]) by groups of cases with noncommunicable diseases, their population controls, and psychiatric comorbidities across any psychiatric disorder, depression, and substance use disorder.

		Premature mortality	Suicide
**Premature mortality**			
**Any psychiatric disorder**			
	**Chronic respiratory diseases**		
	Control, no psychiatric disorder	1.3% [1.3%; 1.3%]	0.1% [0.1%; 0.1%]
	Control, psychiatric disorder	5.2% [5.1%; 5.3%]	0.7% [0.6%; 0.7%]
	Patient, no psychiatric disorder	5.5% [5.4%; 5.6%]	0.1% [0.1%; 0.1%]
	Patient, psychiatric disorder	15.4% [15.1%; 15.8%]	1.2% [1.1%; 1.4%]
	**Cardiovascular diseases**		
	Control, no psychiatric disorder	1.8% [1.8%; 1.8%]	0.1% [0.1%; 0.1%]
	Control, psychiatric disorder	7.2% [7.1%; 7.3%]	0.9% [0.8%; 0.9%]
	Patient, no psychiatric disorder	9.1% [9.0%; 9.2%]	0.1% [0.1%; 0.1%]
	Patient, psychiatric disorder	21.1% [20.7%; 21.4%]	1.6% [1.5%; 1.7%]
	**Diabetes**		
	Control, no psychiatric disorder	1.7% [1.7%; 1.7%]	0.1% [0.1%; 0.1%]
	Control, psychiatric disorder	7.2% [7.0%; 7.3%]	0.8% [0.8%; 0.9%]
	Patient, no psychiatric disorder	7.9% [7.8%; 8.1%]	0.1% [0.1%; 0.1%]
	Patient, psychiatric disorder	16.6% [16.2%; 17.0%]	1.3% [1.2%; 1.5%]
**Depression**			
	**Chronic respiratory diseases**		
	Control, no depression	1.5% [1.5%; 1.6%]	0.1% [0.1%; 0.1%]
	Control, depression	4.5% [4.4%; 4.7%]	0.9% [0.9%; 1.0%]
	Patient, no depression	7.0% [6.9%; 7.1%]	0.2% [0.2%; 0.3%]
	Patient, depression	13.4% [12.8%; 14.0%]	1.7% [1.4%; 1.9%]
	**Cardiovascular diseases**		
	Control, no depression	2.1% [2.1%; 2.1%]	0.1% [0.1%; 0.1%]
	Control, depression	5.9% [5.8%; 6.1%]	1.2% [1.1%; 1.2%]
	Patient, no depression	10.4% [10.4%; 10.5%]	0.3% [0.2%; 0.3%]
	Patient, depression	18.9% [18.3%; 19.4%]	2.2% [2.0%; 2.4%]
	**Diabetes**		
	Control, no depression	2.1% [2%; 2.1%]	0.1% [0.1%; 0.1%]
	Control, depression	5.9% [5.7%; 6.1%]	1.1% [1.0%; 1.1%]
	Patient, no depression	9.2% [9.0%; 9.3%]	0.3% [0.2%; 0.3%]
	Patient, depression	13.5% [12.9%; 14.2%]	1.5% [1.3%; 1.8%]
**Substance use disorder (SUD)**			
	**Chronic respiratory diseases**		
	Control, no SUD	1.4% [1.4%; 1.4%]	0.1% [0.1%; 0.1%]
	Control, SUD	9.6% [9.3%; 9.9%]	1.2% [1.1%; 1.3%]
	Patient, no SUD	6.0% [5.9%; 6.1%]	0.2% [0.2%; 0.2%]
	Patient, SUD	23.3% [22.7%; 24.0%]	2.3% [2.0%; 2.5%]
	**Cardiovascular diseases**		
	Control, no SUD	1.9% [1.9%; 1.9%]	0.1% [0.1%; 0.1%]
	Control, SUD	11.7% [11.5%; 11.8%]	1.3% [1.2%; 1.3%]
	Patient, no SUD	9.7% [9.6%; 9.7%]	0.2% [0.2%; 0.2%]
	Patient, SUD	28.7% [28.2%; 29.3%]	2.4% [2.2%; 2.6%]
	**Diabetes**		
	Control, no SUD	1.8% [1.8%; 1.9%]	0.1% [0.1%; 0.1%]
	Control, SUD	11.9% [11.6%; 12.2%]	1.3% [1.2%; 1.4%]
	Patient, no SUD	8.2% [8.1%; 8.4%]	0.2% [0.2%; 0.2%]
	Patient, SUD	24.2% [23.5%; 24.9%]	2.1% [1.8%; 2.3%]

Differences between groups defined by the combination of noncommunicable disease status (e.g., patients diagnosed with either chronic respiratory diseases, cardiovascular diseases, or diabetes, and controls) and specific psychiatric comorbidity status (e.g., either any psychiatric disorder or depression or substance use disorders) were statistically significant (*P* < 0.001). Direct hypothesis testing of the absolute rate differences between the psychiatric comorbidity categories was not conducted.

Compared to unaffected siblings, we found that patients with any comorbid psychiatric disorder had elevated risks of premature mortality (aHR range: 7.2 to 8.9) and suicide (aHR range: 10.6 to 12.3) relative to patients without psychiatric comorbidities (aHR_PM_ range: 3.0 to 3.9; aHR_S_ range: 1.5 to 1.9) (Table 6). This pattern of associations was similar across specific psychiatric disorders, but with smaller effect sizes for depression (aHR range: 5.3 to 7.4) than substance use disorders (aHR range: 8.3 to 9.9) for (Table 6). We did not find any clear differences in suicide risk between patients with comorbid depression and substance use disorder (aHR range: 9.9 to 13.0; Table 6). Relative to population-wide estimates, we found that the sibling comparison estimates comparing those with comorbid psychiatric disorders with their unaffected siblings were attenuated by at least 25% (S3 Table).

**Table 6:** Hazard ratios of premature mortality and suicide in patients with noncommunicable diseases either with or without comorbid psychiatric disorders compared with sibling controls.

		Crude	Adjusted
		HR [95% CI]	HR [95% CI]
**Premature mortality**			
	**Chronic respiratory diseases**		
	No psychiatric comorbidity	3.0 [2.9; 3.1]	3.0 [2.9; 3.1]
	Any psychiatric comorbidity	7.5 [7.1; 8.1]	7.2 [6.8; 7.7]
	Comorbid depression	5.5 [4.9; 6.1]	5.3 [4.7; 5.9]
	Comorbid substance use disorder	8.6 [7.9; 9.4]	8.3 [7.6; 9.1]
	**Cardiovascular diseases**		
	No psychiatric comorbidity	4.1 [4.0; 4.2]	3.7 [3.6; 3.8]
	Any psychiatric comorbidity	10.2 [9.7; 10.7]	8.9 [8.5; 9.4]
	Comorbid depression	7.7 [7.2; 8.2]	7.4 [7.0; 7.9]
	Comorbid substance use disorder	10.6 [9.9; 11.3]	9.9 [9.3; 10.6]
	**Diabetes**		
	No psychiatric comorbidity	4.0 [3.8; 4.1]	3.9 [3.8; 4.0]
	Any psychiatric comorbidity	8.3 [7.8; 8.9]	7.8 [7.2; 8.3]
	Comorbid depression	6.4 [5.7; 7.3]	6.2 [5.5; 7.0]
	Comorbid substance use disorder	10.4 [9.4; 11.4]	9.7 [8.8; 10.7]
**Suicide**			
	**Chronic respiratory diseases**		
	No psychiatric comorbidity	1.9 [1.7; 2.2]	1.9 [1.6; 2.2]
	Any psychiatric comorbidity	10.8 [8.5; 13.7]	10.6 [8.4; 13.5]
	Comorbid depression	11.0 [8.2; 14.7]	10.7 [8.0; 14.3]
	Comorbid substance use disorder	12.3 [9.4; 16.2]	12.0 [9.2; 15.8]
	**Cardiovascular diseases**		
	No psychiatric comorbidity	1.6 [1.4; 1.8]	1.7 [1.5; 1.9]
	Any psychiatric comorbidity	12.3 [10.2; 14.9]	12.3 [10.2; 15.0]
	Comorbid depression	13.6 [10.9; 16.9]	13.0 [10.9; 16.9]
	Comorbid substance use disorder	11.5 [9.5; 13.9]	10.8 [8.9; 13.1]
	**Diabetes**		
	No psychiatric comorbidity	1.7 [1.4; 2.0]	1.5 [1.3; 1.8]
	Any psychiatric comorbidity	13.4 [9.5; 19.1]	10.8 [8.4; 13.9]
	Comorbid depression	10.5 [7.7; 14.4]	10.0 [7.3; 13.7]
	Comorbid substance use disorder	10.3 [7.2; 14.6]	9.9 [7.0; 14.1]

The estimates are based on stratified Cox regression models where groups of cases and sibling controls were treated as different strata. All models were adjusted for birth year and sex. The adjusted sibling comparison model additionally adjusted for low income and single marital status. Migrant background was controlled for by the design as it does not vary within families. All of the estimates for the comparisons the between patients and sibling controls were statistically significant (*P* < 0.001).

### Comorbid noncommunicable diseases

About 1 in 8 patients (*n =* 141,262) were diagnosed with 2 or all 3 of the examined noncommunicable diseases up to 2013. Of these multimorbid patients, 8.0% (95% CI: 7.8% to 8.1%) died in the 5 years following the onset of their first noncommunicable disease. Compared to their siblings without any of the noncommunicable diseases, those with more than 1 noncommunicable disease were 5 times as likely to die prematurely (aHR: 4.7; 95% CI: 4.6 to 4.9: *P* < 0.001) and 7 times as likely if the medical comorbidity was accompanied by psychiatric comorbidity (aHR: 6.7; 95% CI: 6.3 to 7.2; *P* < 0.001).

### Sensitivity analyses

Complementary sensitivity analyses found that using lifetime diagnoses of any comorbid psychiatric diagnosis (e.g., diagnoses that were given either before or after the onset of the noncommunicable diseases) did not materially change the main findings (S4 Table). We found that type 1 diabetes was associated with higher risks of premature mortality than type 2 diabetes (aHR ranges: 6.4 to 19.4 versus 3.5 to 9.3; S5 Table). Differences between subgroups of substance use disorders were negligible (S6 Table). We found commensurate results when excluding patients diagnosed with 2 or 3 of the noncommunicable diseases from the main analyses (S7 Table), and additional adjustments for BMI in male conscripts did not materially alter our findings (S8 Table). Patients who were hospitalized for their noncommunicable disease at the first presentation typically had higher mortality rates than those in specialist outpatient care (S9 and S10 Tables).

## Discussion

In this longitudinal study of over 1 million patients with chronic respiratory diseases, cardiovascular diseases, and diabetes, we investigated the association of psychiatric comorbidity with premature death in the whole Swedish population over 4 decades. To test the strength of the observed associations, we also used a sibling comparison design to account for potential unmeasured familial confounders. We report 5 main findings:

First, comorbid psychiatric disorder was associated with an increased absolute risk of mortality within 5 years of diagnosis of chronic noncommunicable diseases. Mortality rates ranged between 15% to 21% in individuals with chronic respiratory diseases, cardiovascular diseases, or diabetes who had a comorbid psychiatric disorder. The equivalent risks ranged between 6% to 9% in patients without such comorbidities, representing an absolute risk difference of at least 9%. Similarly, we observed an equivalent absolute risk difference for suicide of at least 1%. In relative terms, we found that patients with comorbid psychiatric disorders were more than twice as likely as patients without such comorbidities to have died prematurely and over 5 times as likely to have died from suicide. These findings therefore extend the findings of an earlier Swedish study [39] that reported strong associations between common psychiatric disorders (e.g., depression and anxiety) and functional limitations in patients with heart failure to mortality outcomes (e.g., all-cause mortality and suicide) in patients with a broader range of cardiovascular diseases. Importantly, these findings highlight the excess mortality burden in countries where psychiatric services for general medical patients are absent or underresourced, and the importance in developing them as part of a comprehensive plan to address the rising challenge of noncommunicable diseases in low- and middle-income countries.

Second, although comorbid depression was associated with an increased absolute rate of mortality, the rate of mortality associated with comorbid substance use disorder was much greater. The 5-year mortality risks was 23% to 29% for patients with comorbid substance use disorder and 13% to 19% in patients with comorbid depression. Compared to their unaffected siblings, we found that the mortality risks were elevated by 8 to 11 times in those with comorbid substance use disorder and by 5 to 7 times in comorbid depression. In contrast, the suicide risk was similar across these comorbidities.

Third, familial factors (genetic and shared early childhood environments) captured through the sibling comparisons, explained between a quarter to a third of the combined effects of the noncommunicable diseases and psychiatric comorbidity. That there remained an elevated risk after adjusting for familial factors provides evidence for the modifiable effects of psychiatric comorbidity on outcomes as it suggests that its effects are not explained solely by a combination of unmeasured familial confounders and measured individual-level confounders (e.g., low income and single marital status). Future observational studies examining the effects of psychiatric comorbidity might consider using family-based research designs to obtain more accurate estimates of the effects of comorbid psychiatric disorders [40].

Fourth, we found that psychiatric comorbidity made a greater contribution to risk of mortality than did somatic multimorbidity. As psychiatric comorbidity is potentially modifiable, this finding has important implications for the priority given to the identification and treatment of comorbid psychiatric disorder when allocating resources and designing services, at least for the medical conditions studied here.

Fifth, by stratifying patients diagnosed with either type 1 or type 2 diabetes, we found that the former was associated with higher premature mortality rates, particularly if it was accompanied by psychiatric comorbidity. The increased mortality rates in type 1 diabetes versus type 2 diabetes has consistently been reported in the literature [41], but the psychiatric comorbidity findings are novel. These findings are expected as type 1 diabetes has typically an adolescent onset with greater disruption of development and chronicity than type 2 diabetes, which usually presents in adulthood [42]. Furthermore, the course of type 1 diabetes is more fluctuating and therefore might lead to psychological difficulties in relation to its control and management. For example, persons with type 1 diabetes could potentially develop chronic anxiety about the risk of experiencing a severe hypoglycemic event [43].

Compared with suicide risk in other common medical conditions, a recent work has reported incident rate ratios of 1.3 for stroke and 1.7 for epilepsy [44], which is lower than the relative risks of the noncommunicable diseases reported in this study. Psychiatric comorbidities are important contributors to deaths by external causes in epilepsy, ranging from adjusted odds ratios of 13 in comorbid depression to 22 in comorbid substance use disorders reported in a nationwide Swedish sibling comparison study [35]. A similar study focusing on suicide risk in traumatic brain injuries reported adjusted odds ratios of at least 15 in patients with comorbid depression and substance use disorders compared to population controls [36]. A population-based Canadian study found that comorbid psychiatric disorders were associated with between 6 to 12 times higher odds of suicide in patients diagnosed with inflammatory bowel disease, multiple sclerosis, and rheumatoid arthritis, compared with population controls [45]. Our findings are therefore broadly consistent with the literature.

Possible mechanisms by which psychiatric comorbidity might increase mortality risks include the association of psychiatric illnesses with smoking, poor nutrition, and lower physical activity [3,46,47]. Substance use disorders are mostly clearly linked with unhealthy lifestyles and may lead to delayed presentation of physical diseases and lower quality of care once physical diseases have been diagnosed [48]. For depression, the association with several inflammatory biomarkers, including CRP [49], could explain elevated mortality risks from natural causes (rather than external ones) [50,51], although the etiological mechanisms for these associations are unclear [52,53]. The metabolic side effects of certain antidepressants and other psychotropic medications, and the increased incidence of type 2 diabetes in those taking antipsychotics, may also explain some of the reported risks, leading to cardiovascular mortality. Antipsychotics are widely prescribed in primary care for a wide range of psychiatric disorders, including depression, anxiety, and personality disorders [54]. In type 1 diabetes, the psychological effects of living with a chronic condition is associated with low mood, which increases risks of depression [55]. This may be more prominent than for type 2 diabetes, where the onset is typically in mid-late adulthood. Mechanisms for suicide risk may include hopelessness about prognosis, effects of physical health on employment and relationships, and the direct effects of mood disorders (via cognitive distortions, negative thinking, and impulsivity). From an etiological viewpoint, comorbid psychiatric disorders and, particularly, substance use disorders, can potentially cause the development of the noncommunicable diseases. Mendelian randomization studies have, for instance, reported that heavy drinking may cause a wide range of cardiometabolic risks [56] and diabetes [57].

Strengths of the current investigation include the use of a large sample facilitated by population-based registers, which allowed us to identify over a million patients with clinically validated diagnoses of 3 noncommunicable diseases of public health importance. The registries further allowed us to match each patient with a maximum of 10 general population controls from data sources with less than 3% attrition, collected in a country with a universal healthcare system, which kept selection bias to a minimum. Prevalence rates of comorbid depression in our study were also comparable to those found by previous research [6,7]. Importantly, this is, to our knowledge, the first study of its kind to have adopted a sibling comparison approach to rigorously account for unmeasured familial confounders.

Among the limitations are that we did not study the effects of psychiatric treatment on mortality outcomes, partly as such information is not available for inpatients and also because it is a different question to the ones we examined. It is possible that treatment of psychiatric disorders, such as depression and substance use disorders, may be less available to people with noncommunicable diseases and psychiatric comorbidities, as the somatic condition overshadows the psychiatric one [4], possibly explaining some of the differences in mortality risk between the different psychiatric disorders. A second limitation is that, by linking population registers, we examined solely cases of psychiatric comorbidities diagnosed in secondary care (outpatient specialist care and inpatient hospitalization). This limitation means that our sample did not include undiagnosed and less severe (primary care) cases, which may mean that the current study likely overestimates the relative effects of psychiatric illness, and underestimate absolute effects. On the other hand, solely selecting patients who present to secondary care is a possible strength because these are the settings that interventions can be most easily provided as liaison psychiatric services could be available, at least to people hospitalized to general medical hospitals. Third, while the sibling comparison approach is a replicated and valid method to account for genetic and environmental factors that are shared between biological full siblings, residual genetic confounding is likely to be present (and can be tested using twin designs). Fourth, due to the nature of nationwide administrative registers, we did not have data on lifestyle factors, such as smoking and physical activities, which could potentially lead to residual confounding. However, the sibling comparison approach allowed us to account for some of the underlying causes of individual differences in such lifestyle factors, including shared early childhood environments and some genetic risks. Fifth is the potential generalizability of our findings to populations outside Sweden. Generalizability is suggested by the similar prevalence rates of the 3 noncommunicable disease studied between Sweden and other high-income countries [58–60]. Comparing rates of comorbid depression across epidemiological studies is difficult because of different measurement tools (e.g., clinical diagnoses versus self-reports), but the rates reported in this investigation are within the range reported in systematic reviews that used structured clinical interviews to measure depression comorbidity [61,62].

One implication of the findings is that they underscore the importance of screening for and treating psychiatric disorders in patients attending medical services with chronic illnesses. For depression, our findings suggest that such screening will identify between 53% to 57% of cases. They also support existing recommendations for screening and treating depression in patients with COPD [63], cardiovascular diseases [64], and diabetes [65]. For alcohol and substance use disorders, our results suggest that such screening can identify between 6% and 8%, a rate twice that of the general population. This suggests that much greater emphasis should be given to screening for and treating these disorders in patients attending medical services [13], perhaps by better integrating psychiatry into them [66]. Although systematic screening may identify psychiatric comorbidities, it will not improve outcome unless it is linked to effective interventions. For example, research in cancer has found that that screening needs to be linked with collaborative depression treatment to improve depression outcomes [67] and that trials need to test outcomes in real-world settings.

Another implication is that prognostic models for mortality in noncommunicable diseases should consider including psychiatric comorbidities. Some cardiovascular risk calculators, such as QRisk3, have added severe mental illness and specific classes of psychotropic medication to their models for future cardiovascular events [68]. However, tools for mortality prognosis do not currently include psychiatric disorders, including one developed using UK Biobank data for the general population [69] and those developed in high-risk hospitalized populations [70,71].

From a public health perspective, 2 possible routes to reducing mortality and morbidity could be considered. First, co-occurring substance use disorder and depression can be overlooked in older persons with other medical problems or not treated. Whether the fragmentation of services, which has tended to locate general/internal medicine, mental health, and substance use disorder services in different locations and under separate managerial and funding arrangements, has contributed to this requires investigation, including examining models where general hospitals directly fund liaison psychiatric and substance services. Attitudes need shifting—based on the understanding that psychiatric illness is not just a “mental health problem” but will influence patient’s survival and is therefore also a “physical health problem.” This will require implementation of a more integrated approach to care [66,72]. There are promising initiatives: for example, in healthcare provided by the Veterans Administration in the US [73]. But there needs to be a clear change in how clinicians, administrators, and public health think about patient care. Second, the findings underscore the potential role of primary care in identifying and treating these comorbidities. Delaying treatment worsens prognosis in mental disorders, and presentation to specialist services for exacerbation of underlying physical health problems may be late in the course of these co-occurring disorders.

Future research can investigate and evaluate screening, improve prognostic modeling, and examine possible mechanisms linking chronic noncommunicable diseases and psychiatric comorbidities, including their bidirectional effects across the life course. More research is also needed to explore the potential etiological contributions of early childhood risk markers using genetically informative research designs. In addition, examining a wider set of medical conditions, including infectious and neurological diseases, would be informative, and test how the pattern and effects of comorbidity vary by underlying condition and the age at onset.

In conclusion, by examining mortality risks in more than 1 million adults, we have found substantial effects of psychiatric comorbidities in patients with cardiac, respiratory, and diabetic conditions. Screening and treatment for co-occurring substance use disorders and depression in these conditions may improve life expectancy.

## Supporting information

S1 Checklist STROBE statement. (DOCX)

S1 Fig Flow charts of the samples used in the study. (PNG)

S1 Table ICD diagnostic codes. (DOCX)

S2 Table One-, 2-, and 5-year cumulative premature mortality and suicide risks by groups of cases with noncommunicable diseases, their population controls, and psychiatric comorbidities across any psychiatric disorder, depression, and substance use disorder. (DOCX)

S3 Table Relative risks of premature mortality and suicide in patients with noncommunicable diseases either with or without comorbid psychiatric disorders compared with population controls. (DOCX)

S4 Table Relative risks of premature mortality and suicide in patients with noncommunicable diseases either with or without comorbid lifetime psychiatric disorders compared with sibling controls. (DOCX)

S5 Table Relative risks of premature mortality and suicide in patients with type 1 or type 2 diabetes either with or without comorbid psychiatric disorders compared with sibling controls. (DOCX)

S6 Table Relative risks of premature mortality and suicide in patients with noncommunicable diseases either with or without comorbid alcohol and drug use disorder use disorders compared with sibling controls. (DOCX)

S7 Table Relative risks of premature mortality and suicide in patients with noncommunicable diseases (excluding those with multiple noncommunicable diseases) either with or without comorbid psychiatric disorders compared with sibling controls. (DOCX)

S8 Table Relative risks of premature mortality and suicide in patients with noncommunicable diseases either with or without comorbid psychiatric disorders compared with sibling controls with additional adjustments for BMI in a subset of male conscripts. (DOCX)

S9 Table Relative risks of premature mortality and suicide in patients with noncommunicable diseases (diagnosed first in inpatient care settings) either with or without comorbid psychiatric disorders compared with sibling controls. (DOCX)

S10 Table Relative risks of premature mortality and suicide in patients with noncommunicable diseases (diagnosed first in outpatient care settings) either with or without comorbid psychiatric disorders compared with sibling controls. (DOCX)

## Abbreviations

aHR: adjusted hazard ratio
BMI: body mass index
cHR: crude hazard ratio
COPD: chronic obstructive pulmonary disease
CR: chronic respiratory diseases
CV: cardiovascular diseases
D: diabetis
ICD: International Classification of Diseases
PPV: positive predictive value
