# Supplementary figures and images for: Psychiatric comorbidity and risk of premature mortality and suicide among those with chronic respiratory diseases, cardiovascular diseases, and diabetes in Sweden: A nationwide matched cohort study of over 1 million patients and their unaffected siblings
Source: PLoS Med. 2022 Jan 27;19(1):e1003864. doi: 10.1371/journal.pmed.1003864 (PMC8794193; doi:10.1371/journal.pmed.1003864)

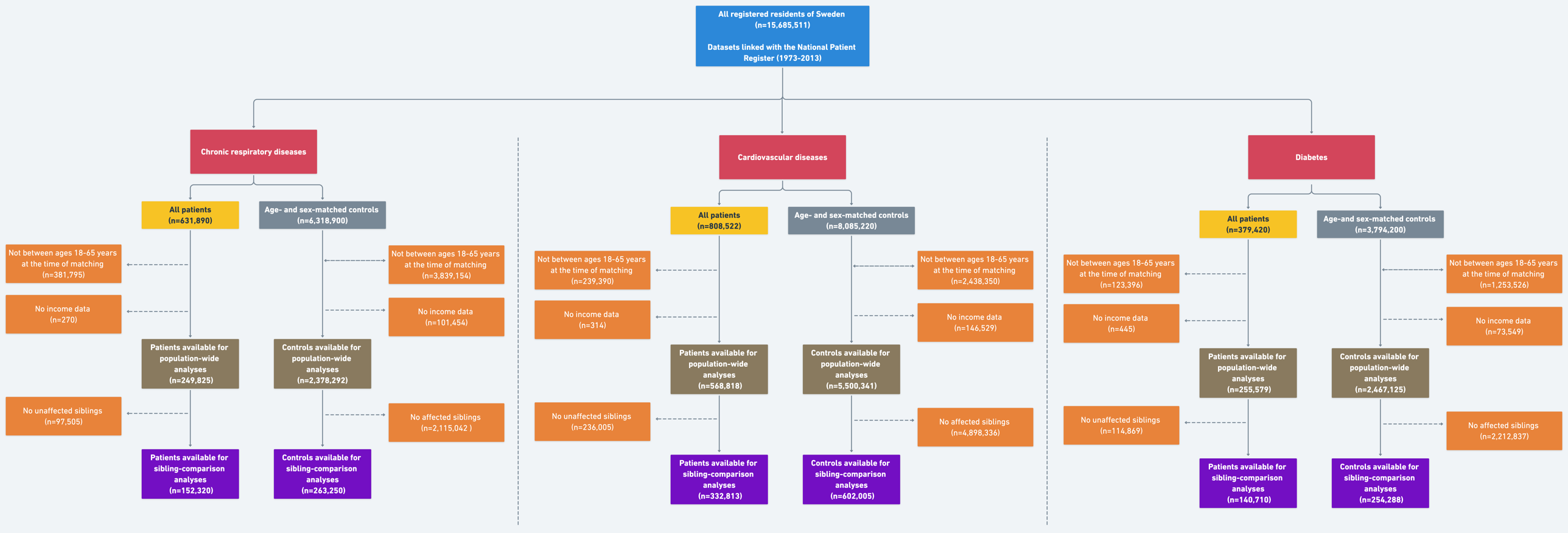

Supplement: S1 Fig — (PNG) [file pmed.1003864.s002.png]
