# Supplementary material for: Psychiatric comorbidity and risk of premature mortality and suicide among those with chronic respiratory diseases, cardiovascular diseases, and diabetes in Sweden: A nationwide matched cohort study of over 1 million patients and their unaffected siblings
Source: PLoS Med. 2022 Jan 27;19(1):e1003864. doi: 10.1371/journal.pmed.1003864 (PMC8794193; doi:10.1371/journal.pmed.1003864)
Supplement: S1 Table — (DOCX) [file pmed.1003864.s003.docx]

**S1 Table. ICD diagnostic codes**

| **Category** | **ICD-8** | **ICD-9** | **ICD-10** |
| --- | --- | --- | --- |
| Chronic respiratory diseases | 490-493 | 490-496 | J40-J47 |
| Cardiovascular diseases | 410-414,  420-429 | 410-417,  420-429 | I20-I52 |
| Diabetes | 250 | 250 | E10-E14 |
| Diabetes, Type 1 | - | - | E10 |
| Diabetes, Type 2 | - | - | E11 |
| Substance use disorder | 291, 292,  303, 304 | 303, 304,  305A, 305X | F10-F14,  F16-F19 |
| Alcohol use disorder | 291, 303 | 303, 305A | F10 |
| Drug use disorder | 292, 304 | 304, 305X | F11-F14, F16-F19 |
| Depression | 296.2, 300.4 | 296B, 300E, 311 | F32-F39, except F32.3 |
| Any psychiatric disorder | 290-315 | 290-319 | F00-F99 |
