# Supplementary material for: Psychiatric comorbidity and risk of premature mortality and suicide among those with chronic respiratory diseases, cardiovascular diseases, and diabetes in Sweden: A nationwide matched cohort study of over 1 million patients and their unaffected siblings
Source: PLoS Med. 2022 Jan 27;19(1):e1003864. doi: 10.1371/journal.pmed.1003864 (PMC8794193; doi:10.1371/journal.pmed.1003864)
Supplement: S2 Table — (DOCX) [file pmed.1003864.s004.docx]

**S2 Table. 1-, 2-, and 5-year cumulative premature mortality and suicide risks by groups of cases with non-communicable diseases, their population controls, and psychiatric comorbidities across any psychiatric disorder, depression, and substance use disorder**

|  |  | **1 year** | **2 year** | **5 year** |
| --- | --- | --- | --- | --- |
| **Premature mortality** |  |  |  |  |
|  |  |  |  |  |
| **Any psychiatric disorder** |  |  |  |  |
|  | **Chronic respiratory diseases** |  |  |  |
|  | Control, no psychiatric disorder | 0.25% [0.24%; 0.25%] | 0.50% [0.49%; 0.51%] | 1.31% [1.29%; 1.33%] |
|  | Control, psychiatric disorder | 1.00% [0.96%; 1.05%] | 2.00% [1.94%; 2.06%] | 5.17% [5.05%; 5.28%] |
|  | Patient, no psychiatric disorder | 2.05% [1.99%; 2.11%] | 3.05% [2.97%; 3.13%] | 5.47% [5.36%; 5.58%] |
|  | Patient, psychiatric disorder | 5.36% [5.17%; 5.56%] | 8.35% [8.10%; 8.60%] | 15.43% [15.07%; 15.79%] |
|  |  |  |  |  |
|  | **Cardiovascular diseases** |  |  |  |
|  | Control, no psychiatric disorder | 0.32% [0.32%; 0.33%] | 0.67% [0.66%; 0.68%] | 1.80% [1.79%; 1.81%] |
|  | Control, psychiatric disorder | 1.43% [1.39%; 1.46%] | 2.85% [2.80%; 2.90%] | 7.18% [7.09%; 7.27%] |
|  | Patient, no psychiatric disorder | 5.13% [5.07%; 5.19%] | 6.33% [6.26%; 6.40%] | 9.09% [9.01%; 9.18%] |
|  | Patient, psychiatric disorder | 11.28% [11.06%; 11.49%] | 14.15% [13.91%; 14.39%] | 21.05% [20.75%; 21.36%] |
|  |  |  |  |  |
|  | **Diabetes** |  |  |  |
|  | Control, no psychiatric disorder | 0.32% [0.32%; 0.33%] | 0.66% [0.65%; 0.67%] | 1.71% [1.70%; 1.73%] |
|  | Control, psychiatric disorder | 1.45% [1.40%; 1.51%] | 2.93% [2.85%; 3.00%] | 7.18% [7.04%; 7.31%] |
|  | Patient, no psychiatric disorder | 3.09% [3.02%; 3.17%] | 4.50% [4.41%; 4.60%] | 7.94% [7.81%; 8.07%] |
|  | Patient, psychiatric disorder | 5.36% [5.14%; 5.57%] | 8.40% [8.13%; 8.67%] | 16.61% [16.21%; 17.01%] |
|  |  |  |  |  |
| **Depression** |  |  |  |  |
|  | **Chronic respiratory diseases** |  |  |  |
|  | Control, no depression | 0.30% [0.29%; 0.30%] | 0.60% [0.59%; 0.61%] | 1.54% [1.53%; 1.56%] |
|  | Control, depression | 0.87% [0.80%; 0.94%] | 1.76% [1.66%; 1.86%] | 4.54% [4.35%; 4.74%] |
|  | Patient, no depression | 2.63% [2.56%; 2.69%] | 3.92% [3.84%; 4.01%] | 7.01% [6.89%; 7.12%] |
|  | Patient, depression | 4.12% [3.82%; 4.41%] | 6.90% [6.50%; 7.29%] | 13.38% [12.79%; 13.98%] |
|  |  |  |  |  |
|  | **Cardiovascular diseases** |  |  |  |
|  | Control, no depression | 0.39% [0.39%; 0.40%] | 0.81% [0.80%; 0.82%] | 2.13% [2.12%; 2.15%] |
|  | Control, depression | 1.20% [1.14%; 1.26%] | 2.36% [2.28%; 2.44%] | 5.92% [5.78%; 6.07%] |
|  | Patient, no depression | 5.86% [5.80%; 5.93%] | 7.24% [7.17%; 7.31%] | 10.45% [10.36%; 10.53%] |
|  | Patient, depression | 9.91% [9.54%; 10.27%] | 12.64% [12.22%; 13.06%] | 18.88% [18.34%; 19.42%] |
|  |  |  |  |  |
|  | **Diabetes** |  |  |  |
|  | Control, no depression | 0.40% [0.39%; 0.41%] | 0.80% [0.79%; 0.82%] | 2.05% [2.03%; 2.07%] |
|  | Control, depression | 1.22% [1.13%; 1.30%] | 2.50% [2.38%; 2.63%] | 5.87% [5.65%; 6.08%] |
|  | Patient, no depression | 3.41% [3.34%; 3.49%] | 5.06% [4.96%; 5.15%] | 9.15% [9.02%; 9.28%] |
|  | Patient, depression | 4.63% [4.28%; 4.98%] | 7.16% [6.72%; 7.61%] | 13.53% [12.86%; 14.19%] |
| **Substance use disorder (SUD)** |  |  |  |  |
|  | **Chronic respiratory diseases** |  |  |  |
|  | Control, no SUD | 0.27% [0.26%; 0.28%] | 0.55% [0.54%; 0.56%] | 1.42% [1.40%; 1.44%] |
|  | Control, SUD | 1.90% [1.79%; 2.01%] | 3.76% [3.60%; 3.92%] | 9.58% [9.31%; 9.86%] |
|  | Patient, no SUD | 2.23% [2.17%; 2.29%] | 3.36% [3.28%; 3.43%] | 6.02% [5.91%; 6.13%] |
|  | Patient, SUD | 8.26% [7.88%; 8.64%] | 12.72% [12.25%; 13.2%] | 23.33% [22.67%; 23.99%] |
|  |  |  |  |  |
|  | **Cardiovascular diseases** |  |  |  |
|  | Control, no SUD | 0.35% [0.34%; 0.35%] | 0.72% [0.72%; 0.73%] | 1.93% [1.91%; 1.94%] |
|  | Control, SUD | 2.39% [2.32%; 2.47%] | 4.76% [4.65%; 4.86%] | 11.66% [11.48%; 11.84%] |
|  | Patient, no SUD | 5.47% [5.41%; 5.54%] | 6.75% [6.68%; 6.82%] | 9.66% [9.58%; 9.75%] |
|  | Patient, SUD | 14.94% [14.56%; 15.32%] | 18.92% [18.5%; 19.35%] | 28.73% [28.21%; 29.26%] |
|  |  |  |  |  |
|  | **Diabetes** |  |  |  |
|  | Control, no SUD | 0.36% [0.35%; 0.36%] | 0.72% [0.71%; 0.73%] | 1.85% [1.83%; 1.87%] |
|  | Control, SUD | 2.42% [2.31%; 2.53%] | 4.95% [4.79%; 5.12%] | 11.89% [11.62%; 12.16%] |
|  | Patient, no SUD | 3.20% [3.13%; 3.27%] | 4.66% [4.57%; 4.75%] | 8.25% [8.13%; 8.37%] |
|  | Patient, SUD | 7.28% [6.89%; 7.66%] | 11.93% [11.43%; 12.42%] | 24.21% [23.49%; 24.92%] |
| **Suicide** |  |  |  |  |
|  |  |  |  |  |
| **Any psychiatric disorder** |  |  |  |  |
|  | **Chronic respiratory diseases** |  |  |  |
|  | Control, no psychiatric disorder | 0.01% [0.01%; 0.01%] | 0.02% [0.02%; 0.02%] | 0.06% [0.06%; 0.07%] |
|  | Control, psychiatric disorder | 0.13% [0.12%; 0.15%] | 0.28% [0.25%; 0.30%] | 0.66% [0.62%; 0.70%] |
|  | Patient, no psychiatric disorder | 0.03% [0.02%; 0.04%] | 0.05% [0.04%; 0.06%] | 0.13% [0.11%; 0.15%] |
|  | Patient, psychiatric disorder | 0.33% [0.28%; 0.38%] | 0.60% [0.53%; 0.67%] | 1.25% [1.13%; 1.36%] |
|  |  |  |  |  |
|  | **Cardiovascular diseases** |  |  |  |
|  | Control, no psychiatric disorder | 0.01% [0.01%; 0.01%] | 0.03% [0.03%; 0.03%] | 0.08% [0.08%; 0.08%] |
|  | Control, psychiatric disorder | 0.18% [0.17%; 0.20%] | 0.35% [0.33%; 0.37%] | 0.85% [0.82%; 0.89%] |
|  | Patient, no psychiatric disorder | 0.05% [0.04%; 0.06%] | 0.07% [0.07%; 0.08%] | 0.14% [0.13%; 0.15%] |
|  | Patient, psychiatric disorder | 0.75% [0.69%; 0.81%] | 0.98% [0.91%; 1.05%] | 1.58% [1.48%; 1.67%] |
|  |  |  |  |  |
|  | **Diabetes** |  |  |  |
|  | Control, no psychiatric disorder | 0.01% [0.01%; 0.02%] | 0.03% [0.03%; 0.03%] | 0.08% [0.08%; 0.09%] |
|  | Control, psychiatric disorder | 0.19% [0.17%; 0.21%] | 0.35% [0.32%; 0.38%] | 0.83% [0.78%; 0.88%] |
|  | Patient, no psychiatric disorder | 0.03% [0.02%; 0.03%] | 0.05% [0.04%; 0.06%] | 0.13% [0.11%; 0.15%] |
|  | Patient, psychiatric disorder | 0.33% [0.28%; 0.39%] | 0.56% [0.49%; 0.64%] | 1.33% [1.20%; 1.46%] |
|  |  |  |  |  |
| **Depression** |  |  |  |  |
|  | **Chronic respiratory diseases** |  |  |  |
|  | Control, no depression | 0.02% [0.01%; 0.02%] | 0.03% [0.03%; 0.03%] | 0.09% [0.08%; 0.09%] |
|  | Control, depression | 0.19% [0.15%; 0.22%] | 0.40% [0.35%; 0.45%] | 0.94% [0.85%; 1.03%] |
|  | Patient, no depression | 0.06% [0.05%; 0.07%] | 0.11% [0.09%; 0.12%] | 0.25% [0.22%; 0.27%] |
|  | Patient, depression | 0.43% [0.33%; 0.53%] | 0.84% [0.70%; 0.99%] | 1.68% [1.44%; 1.91%] |
|  |  |  |  |  |
|  | **Cardiovascular diseases** |  |  |  |
|  | Control, no depression | 0.02% [0.02%; 0.02%] | 0.04% [0.04%; 0.05%] | 0.12% [0.11%; 0.12%] |
|  | Control, depression | 0.26% [0.23%; 0.29%] | 0.48% [0.44%; 0.51%] | 1.16% [1.10%; 1.23%] |
|  | Patient, no depression | 0.10% [0.09%; 0.11%] | 0.14% [0.13%; 0.15%] | 0.25% [0.24%; 0.27%] |
|  | Patient, depression | 1.20% [1.06%; 1.33%] | 1.55% [1.39%; 1.71%] | 2.23% [2.02%; 2.44%] |
|  |  |  |  |  |
|  | **Diabetes** |  |  |  |
|  | Control, no depression | 0.02% [0.02%; 0.02%] | 0.05% [0.04%; 0.05%] | 0.12% [0.11%; 0.12%] |
|  | Control, depression | 0.25% [0.21%; 0.28%] | 0.46% [0.41%; 0.52%] | 1.05% [0.96%; 1.15%] |
|  | Patient, no depression | 0.05% [0.04%; 0.06%] | 0.1% [0.09%; 0.11%] | 0.26% [0.24%; 0.28%] |
|  | Patient, depression | 0.52% [0.40%; 0.65%] | 0.77% [0.61%; 0.92%] | 1.52% [1.27%; 1.77%] |
| **Substance use disorder (SUD)** |  |  |  |  |
|  | **Chronic respiratory diseases** |  |  |  |
|  | Control, no SUD | 0.01% [0.01%; 0.02%] | 0.03% [0.03%; 0.03%] | 0.08% [0.08%; 0.09%] |
|  | Control, SUD | 0.25% [0.21%; 0.29%] | 0.50% [0.44%; 0.56%] | 1.16% [1.06%; 1.26%] |
|  | Patient, no SUD | 0.04% [0.04%; 0.05%] | 0.08% [0.07%; 0.09%] | 0.18% [0.16%; 0.20%] |
|  | Patient, SUD | 0.62% [0.50%; 0.73%] | 1.10% [0.94%; 1.25%] | 2.29% [2.04%; 2.55%] |
|  |  |  |  |  |
|  | **Cardiovascular diseases** |  |  |  |
|  | Control, no SUD | 0.02% [0.02%; 0.02%] | 0.04% [0.04%; 0.04%] | 0.11% [0.1%; 0.11%] |
|  | Control, SUD | 0.29% [0.26%; 0.32%] | 0.55% [0.51%; 0.59%] | 1.28% [1.22%; 1.34%] |
|  | Patient, no SUD | 0.09% [0.08%; 0.10%] | 0.13% [0.12%; 0.14%] | 0.21% [0.2%; 0.23%] |
|  | Patient, SUD | 1.12% [1.00%; 1.23%] | 1.49% [1.36%; 1.63%] | 2.44% [2.25%; 2.63%] |
|  |  |  |  |  |
|  | **Diabetes** |  |  |  |
|  | Control, no SUD | 0.02% [0.02%; 0.02%] | 0.04% [0.04%; 0.04%] | 0.11% [0.10%; 0.11%] |
|  | Control, SUD | 0.31% [0.27%; 0.35%] | 0.55% [0.49%; 0.60%] | 1.34% [1.24%; 1.44%] |
|  | Patient, no SUD | 0.05% [0.04%; 0.06%] | 0.08% [0.07%; 0.10%] | 0.20% [0.18%; 0.22%] |
|  | Patient, SUD | 0.48% [0.37%; 0.58%] | 0.87% [0.72%; 1.02%] | 2.08% [1.83%; 2.34%] |

*Notes: Differences between groups defined by the combination of non-communicable disease status (e.g., patients diagnosed with either chronic respiratory diseases, cardiovascular diseases or diabetes, and controls) and specific psychiatric comorbidity status (e.g., either any psychiatric disorder or depression or substance use disorders) were statistically significant (P<0.001). Direct hypothesis testing of the absolute rate differences between the psychiatric comorbidity categories was not conducted.*
