# Supplementary material for: Psychiatric comorbidity and risk of premature mortality and suicide among those with chronic respiratory diseases, cardiovascular diseases, and diabetes in Sweden: A nationwide matched cohort study of over 1 million patients and their unaffected siblings
Source: PLoS Med. 2022 Jan 27;19(1):e1003864. doi: 10.1371/journal.pmed.1003864 (PMC8794193; doi:10.1371/journal.pmed.1003864)
Supplement: S3 Table — (DOCX) [file pmed.1003864.s005.docx]

**S3 Table. Relative risks of premature mortality and suicide in patients with non-communicable diseases either with or without comorbid psychiatric disorders compared with population controls**

|  |  | **Patients versus**  **population controls** | **Patients versus**  **sibling controls (Reference)** | |
| --- | --- | --- | --- | --- |
|  |  | **Crude** | **Crude** | **Adjusted** |
|  |  | **HR [95% CI]** | **HR [95% CI]** | **HR [95% CI]** |
| **Premature mortality** |  |  |  |  |
|  | **Chronic respiratory diseases** |  |  |  |
|  | No psychiatric comorbidity | 3.6 [3.5; 3.6] | 3.0 [2.9; 3.1] | 3.0 [2.9; 3.1] |
|  | Any psychiatric comorbidity | 10.7 [10.4; 11.0] | 7.5 [7.1; 8.1] | 7.2 [6.8; 7.7] |
|  | Comorbid depression | 8.2 [7.7; 8.6] | 5.5 [4.9; 6.1] | 5.3 [4.7; 5.9] |
|  | Comorbid substance use disorder | 13.5 [13.0; 14.1] | 8.6 [7.9; 9.4] | 8.3 [7.6; 9.1] |
|  |  |  |  |  |
|  | **Cardiovascular diseases** |  |  |  |
|  | No psychiatric comorbidity | 4.1 [4.0; 4.1] | 4.1 [4.0; 4.2] | 3.7 [3.6; 3.8] |
|  | Any psychiatric comorbidity | 12.1 [11.8; 12.3] | 10.2 [9.7; 10.7] | 8.9 [8.5; 9.4] |
|  | Comorbid depression | 9.3 [8.9; 9.6] | 7.7 [7.2; 8.2] | 7.4 [7.0; 7.9] |
|  | Comorbid substance use disorder | 14.5 [14.1; 14.9] | 10.6 [9.9; 11.3] | 9.9 [9.3; 10.6] |
|  |  |  |  |  |
|  | **Diabetes** |  |  |  |
|  | No psychiatric comorbidity | 4.6 [4.6; 4.7] | 4.0 [3.8; 4.1] | 3.9 [3.8; 4.0] |
|  | Any psychiatric comorbidity | 10.4 [10.1; 10.7] | 8.3 [7.8; 8.9] | 7.8 [7.2; 8.3] |
|  | Comorbid depression | 7.3 [6.9; 7.7] | 6.4 [5.7; 7.3] | 6.2 [5.5; 7.0] |
|  | Comorbid substance use disorder | 13.4 [12.9; 13.9] | 10.4 [9.4; 11.4] | 9.7 [8.8; 10.7] |
| **Suicide** |  |  |  |  |
|  | **Chronic respiratory diseases** |  |  |  |
|  | No psychiatric comorbidity | 2.0 [1.8; 2.2] | 1.9 [1.7; 2.2] | 1.9 [1.6; 2.2] |
|  | Any psychiatric comorbidity | 17.6 [15.6; 19.7] | 10.8 [8.5; 13.7] | 10.6 [8.4; 13.5] |
|  | Comorbid depression | 19.7 [16.3; 23.8] | 11.0 [8.2; 14.7] | 10.7 [8.0; 14.3] |
|  | Comorbid substance use disorder | 23.0 [20.6; 25.6] | 12.3 [9.4; 16.2] | 12.0 [9.2; 15.8] |
|  |  |  |  |  |
|  | **Cardiovascular diseases** |  |  |  |
|  | No psychiatric comorbidity | 1.6 [1.5; 1.7] | 1.6 [1.4; 1.8] | 1.7 [1.5-1.9] |
|  | Any psychiatric comorbidity | 19.1 [17.6; 20.7] | 12.3 [10.2; 14.9] | 12.3 [10.2; 15.0] |
|  | Comorbid depression | 21.4 [19.8; 23.2] | 13.6 [10.9; 16.9] | 13.0 [10.9; 16.9] |
|  | Comorbid substance use disorder | 18.5 [17.3; 19.8] | 11.5 [9.5; 13.9] | 10.8 [8.9; 13.1] |
|  |  |  |  |  |
|  | **Diabetes** |  |  |  |
|  | No psychiatric comorbidity | 1.7 [1.5; 1.9] | 1.7 [1.4; 2.0] | 1.5 [1.3; 1.8] |
|  | Any psychiatric comorbidity | 13.6 [12.1; 15.2] | 13.4 [9.5; 19.1] | 10.8 [8.4; 13.9] |
|  | Comorbid depression | 13.2 [10.9; 16.1] | 10.5 [7.7; 14.4] | 10.0 [7.3; 13.7] |
|  | Comorbid substance use disorder | 13.7 [11.8; 15.9] | 10.3 [7.2; 14.6] | 9.9 [7.0; 14.1] |

*Notes: The estimates are based on stratified Cox regression models where groups of cases and controls (matched on sex and birth year) were treated as different strata. All biological full-siblings were included as controls in the sibling models, which were adjusted for birth year and sex. The adjusted sibling-comparison model additionally accounted for low income and single marital status. Migrant background was controlled for by the design as it does not vary within families. The sample sizes varied across chronic respiratory diseases (n_patients vs population controls_=2,628,117; n_patients vs sibling controls_=*415,570*), cardiovascular diseases (n_patients vs population controls_=*6,069,159*; n_patients vs sibling controls_=*934,818*), and diabetes (n_patients vs population controls_=*2,722,704*; n_patients vs sibling controls_=*394,998*). All of the estimates for the comparisons the between patients and either the population or sibling controls were statistically significant (P<0.001).*
