# Supplementary material for: Psychiatric comorbidity and risk of premature mortality and suicide among those with chronic respiratory diseases, cardiovascular diseases, and diabetes in Sweden: A nationwide matched cohort study of over 1 million patients and their unaffected siblings
Source: PLoS Med. 2022 Jan 27;19(1):e1003864. doi: 10.1371/journal.pmed.1003864 (PMC8794193; doi:10.1371/journal.pmed.1003864)
Supplement: S4 Table — (DOCX) [file pmed.1003864.s006.docx]

**S4 Table. Relative risks of premature mortality and suicide in patients with non-communicable diseases either with or without comorbid lifetime psychiatric disorders compared with sibling controls**

|  |  | **Lifetime psychiatric comorbidity** | | **Pre-existing psychiatric comorbidities (Reference)** | |
| --- | --- | --- | --- | --- | --- |
|  |  | **Crude** | **Adjusted** | **Crude** | **Adjusted** |
|  |  | **HR [95% CI]** | **HR [95% CI]** | **HR [95% CI]** | **HR [95% CI]** |
| **Premature mortality** |  |  |  |  |  |
|  | **Chronic respiratory diseases** |  |  |  |  |
|  | No psychiatric comorbidity | 3.3 [3.2; 3.5] | 3.3 [3.2; 3.5] | 3.0 [2.9; 3.1] | 3.0 [2.9; 3.1] |
|  | Any psychiatric comorbidity | 6.0 [5.6; 6.3] | 5.7 [5.4; 6.1] | 7.5 [7.1; 8.1] | 7.2 [6.8; 7.7] |
|  | **Cardiovascular diseases** |  |  |  |  |
|  | No psychiatric comorbidity | 4.2 [4.1; 4.3] | 4.2 [4.1; 4.3] | 4.1 [4.0; 4.2] | 3.7 [3.6; 3.8] |
|  | Any psychiatric comorbidity | 6.2 [6.0; 6.5] | 5.9 [5.7; 6.1] | 10.2 [9.7; 10.7] | 8.9 [8.5; 9.4] |
|  | **Diabetes** |  |  |  |  |
|  | No psychiatric comorbidity | 4.3 [4.1; 4.4] | 4.2 [4.0; 4.4] | 4.0 [3.8; 4.1] | 3.9 [3.8; 4.0] |
|  | Any psychiatric comorbidity | 6.9 [6.5; 7.3] | 6.5 [6.2; 6.9] | 8.3 [7.8; 8.9] | 7.8 [7.2; 8.3] |
| **Suicide** |  |  |  |  |  |
|  | **Chronic respiratory diseases** |  |  |  |  |
|  | No psychiatric comorbidity | 1.8 [1.6; 2.1] | 1.9 [1.7; 2.2] | 1.9 [1.7; 2.2] | 1.9 [1.6; 2.2] |
|  | Any psychiatric comorbidity | 9.8 [7.9; 12.3] | 9.7 [7.8; 12.2] | 10.8 [8.5; 13.7] | 10.6 [8.4; 13.5] |
|  | **Cardiovascular diseases** |  |  |  |  |
|  | No psychiatric comorbidity | 1.9 [1.7; 2.1] | 1.9 [1.7; 2.1] | 1.6 [1.4; 1.8] | 1.7 [1.5-1.9] |
|  | Any psychiatric comorbidity | 11.1 [9.5; 13.1] | 10.8 [9.2; 12.7] | 12.3 [10.2; 14.9] | 12.3 [10.2; 15.0] |
|  | **Diabetes** |  |  |  |  |
|  | No psychiatric comorbidity | 2.0 [1.6; 2.6] | 2.0 [1.6; 2.5] | 1.7 [1.4; 2.0] | 1.5 [1.3; 1.8] |
|  | Any psychiatric comorbidity | 9.1 [7.2; 11.5] | 9.0 [7.1; 11.3] | 13.4 [9.5; 19.1] | 10.8 [8.4; 13.9] |

*Notes: The estimates are based on stratified Cox regression models where groups of cases and sibling controls were treated as different strata. All models were adjusted for birth year and sex. The adjusted sibling-comparison model additionally accounted for low income and single marital status. Migrant background was controlled for by the design as it does not vary within families. The sample sizes varied across chronic respiratory diseases (n=415,570), cardiovascular diseases (n=934,818), and diabetes (n=394,998). All of the estimates for the comparisons the between patients and sibling controls were statistically significant (P<0.001).*
